# Instantaneous formation of SiO_x_ nanocomposite for high capacity lithium ion batteries by enhanced disproportionation reaction during plasma spray physical vapor deposition

**DOI:** 10.1080/14686996.2016.1240574

**Published:** 2016-11-09

**Authors:** Tohru Tashiro, Masashi Dougakiuchi, Makoto Kambara

**Affiliations:** ^a^ Department of Materials Engineering, The University of Tokyo, Hongo, Japan; ^b^ Shimane Institute for Industrial Technology, Matsue, Japan

**Keywords:** Lithium ion batteries, silicon monoxide nanoparticle, disproportionation reaction, plasma spray, 50 Energy materials, 103 Composites, 206 Energy conversion / transport / storage / recovery, 305 Plasma / Laser processing

## Abstract

Nanocomposite SiO_x_ particles have been produced by a single step plasma spray physical vapor deposition (PS-PVD) through rapid condensation of SiO vapors and the subsequent disproportionation reaction. Core-shell nanoparticles, in which 15 nm crystalline Si is embedded within the amorphous SiO_x_ matrix, form under typical PS-PVD conditions, while 10 nm amorphous particles are formed when processed with an increased degree of non-equilibrium effect. Addition of CH_4_ promotes reduction in the oxygen content *x* of SiO_*x*_, and thereby increases the Si volume in a nanocomposite particle. As a result, core-shell nanoparticles with *x* = 0.46 as anode exhibit increased initial efficiency and the capacity of lithium ion batteries while maintaining cyclability. Furthermore, it is revealed that the disproportionation reaction of SiO is promoted in nanosized particles attaining increased Si diffusivity by two orders of magnitude compared to that in bulk, which facilitates instantaneous composite nanoparticle formation during PS-PVD.

## Graphical abstract

**Figure:**
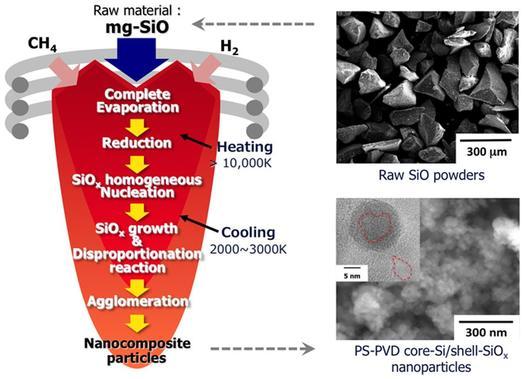

## 1. Introduction

Silicon is a strong candidate for anodes in high density lithium ion batteries (LIB) owing to its 10-fold higher theoretical gravimetric and volumetric capacities than those of the conventional carbonous material. To make the best of its potential, however, structuring the material is necessary to cope with fracturing associated with a huge volume change as a result of (de)lithiation during (dis)charge reaction. Various structures have been reported to be effective in maintaining high cycle capacity:[1–10] decreasing the size of the material improves the capacity at longer cycles,[1–3] and in particular sizes smaller than 150 nm are reported to be free from fracturing.[4] However, smaller particles could reduce the initial efficiency as a result of the increased solid electrolyte interphase (SEI) formation on the increased specific surface area. Coating the silicon materials with carbonous materials works to improve the cyclability, possibly due to more stable SEI formation.[5,6] Composites including porous and nanotube structures are also effective in accommodating the Si dilation during lithiation and maintaining the contacts between particles and current collector after delithiation.**[** 7–10] Another approach is to use SiO as active material.[11–21] Using the characteristic disproportionation reaction of SiO, nanosized Si can be dispersed within a SiO_2_ matrix that is expected to reinforce the Si crystalline precipitates and attain stable cyclability for long cycles.[12–14] Amorphous Si (a-Si) could also form by disproportionation reaction at temperatures lower than 800°C,[22] such that more stable cyclability can be anticipated,[23–25] although a-Si formation requires in general an annealing time longer than several hours. In addition, the presence of oxygen in this material becomes a source of irreversible capacity and the large initial capacity drop is a critical issue of this material when designing the battery structure.[20] Reduction of SiO is thus important not only to improve the initial efficiency but also to increase the volumetric capacity as a result of the increased relative volume of the Si active material. However, conventional annealing under a reducing atmosphere is not effective enough to attain reduction uniformly in a bulk SiO.

Plasma spray physical vapor deposition (PS-PVD) is a potential approach in producing composite SiO nanoparticles (SiO-NP) and reducing the oxygen content at the same time. The elemental process of SiO-NP formation in PS-PVD is evaporation of SiO raw powders and the subsequent condensation of these vapors. Therefore, addition of CH_4_ to SiO at the vapor state promotes removal of oxygen from the vapor mixture owing to preferable formation of CO than SiO_2_ at temperatures higher than 1900 K,[26] and the oxygen content of the SiO-NP so produced after condensation is expected to decrease accordingly. In addition, SiO-NP undergoes disproportionation reaction immediately after condensation to form crystalline Si core structure. SiO-NP produced by PS-PVD in industry compatible high-throughput production using metallurgy grade SiO raw powders have shown a clear increase in capacity while maintaining cyclability.[27] This characteristic process of PS-PVD further implies that the SiO-NP structure can be modified by tuning the cooling history during co-condensation of SiO-CH_4_ vapor mixtures. Especially with large non-equilibrium effect by rapid gas quenching, together with CH_4_ addition, a-Si core structure is expected to form by low temperature disproportionation reaction in smaller SiO-NP with reduced oxygen content. In this article, we report the advantages and uniqueness of PS-PVD SiO-NP as anode for an increase in the capacity and cyclability of LIB, and also the fundamental path of composite formation during PS-PVD with particular emphasis on the enhanced disproportionation reaction in nanosized particles.

## 2. Experimental details

PS-PVD has been carried out with a hybrid PS system. Compared with the conventional PS systems, a greater degree of complete evaporation of coarse raw powders is anticipated due to a direct injection of powders into the highest temperature region of the direct-current (DC) plasma jet superimposing the radio-frequency (RF) plasma flame, which is advantageous for higher production throughputs.[28,29] Metallurgical grade SiO powders are used as raw materials and are injected into the plasma jet generated with Ar and H_2_. A water-cooled gas-quenching vessel is placed underneath the plasma torch so that the plasma gas including the evaporated SiO vapors is immediately cooled and the nanoparticles so formed after condensation are attached on the vessel walls. Schematics of the system can be found elsewhere.[30] To modify the nanoparticle structures, two plasma conditions, at which different powder heating and cooling histories are expected, are employed; condition [A] as the typical PS-PVD and [B] for an increased quenching capability. The detailed plasma conditions are listed in Table 1. In brief, for case [B], plasma is generated at slightly higher pressure of 500 Torr with an increased RF input power in a smaller plasma torch tube. Under this condition, according to the fluid dynamics simulation,[31] the high temperature gas jet is confined axially and the cooler surrounding gas is caught more significantly at the tail flame in the gas-quenching vessel, resulting in a rather rapid gas cooling speed especially near the nucleation and growth temperature of SiO. For comparison, CH_4_ gas is also added at a fixed molar ratio of C/Si = 0.25, at which no SiC phase was detected in the previous work.[27]

**Table 1.** PS-PVD conditions.

Parameter	Case [A]		Case [B]
DC power (kW)	8		9
RF power (kW)	90		100
Pressure (Torr)	400		500
DC Ar flow rate (slm)	10		
Radial Ar flow rate (slm)		140	
Tangential Ar flow rate (slm)		30	
Powder carrier Ar flow rate (slm)		3.6	
Radial H2 flow rate (slm)		30	
CH4 addition (CH4/SiO molar ratio)	0, 0.25		
ICP torch dia. and length (mm)	60, 150		40,100
SiO powder average size (μm)	165		15
Powder feeding rate (g min–1)	8.0		2.2
Processing time (min)		10	

Abbreviations: ICP, inductively coupled plasma; slm, standard liter per minute.

The PS-PVD particles collected from the vessel are ground in a mortar and sieved with a 20 μm mesh to remove greatly agglomerated particles. The existing phases are analyzed by X-ray diffraction (XRD) with Cu Kα irradiation. The XRD patterns are also used for Rietveld analysis to evaluate the crystallite size and the relative amount of the phases involved in the particles. Raman scattering is used to identify the presence of the amorphous structure in the PS-PVD particles. The nanostructure of the particles is observed by scanning transmission electron microscopy (STEM) and field-emission scanning electron microscopy (FE-SEM). The oxygen content of the PS-PVD particles is evaluated from the peak area fraction of XRD patterns after Rietveld analysis, assuming that the broad peak is attributed to SiO_2_ phase. For the particles with no crystalline structure, thermogravimetry (TG) is employed and the residual oxygen amount is estimated by the increased weight after annealing under oxygen flow atmosphere with an assumption of complete oxidation to form SiO_2_.

The PS-PVD particles after sieving are mixed with a conducting regent and polyimide binder at a fixed weight ratio of 60:15:25 to form a slurry and they are applied to a Cu foil current collector with a thickness of 40 μm. After roll-pressing and drying in Ar flow at 110 °C for 2 h, a 15 mm diameter anode is prepared. A half-coin cell using lithium metal as counter electrode is assembled with the electrolyte of LiPF_6_ dissolved in ethylene carbonate (EC) and diethyl carbonate (DEC) mixture solvent with EC:DEC = 1:1 vol. The battery cycle test is carried out at a fixed temperature of 23 °C under a constant current mode with 0.1 mA (corresponding to 0.02 C rate) for the first three cycles and 0.5 mA (0.1 C) for the remaining cycles.

## 3. Results and discussion

### 3.1. Characteristics of Si/SiO_x_ nanoparticles and battery performance

**Figure 1.**
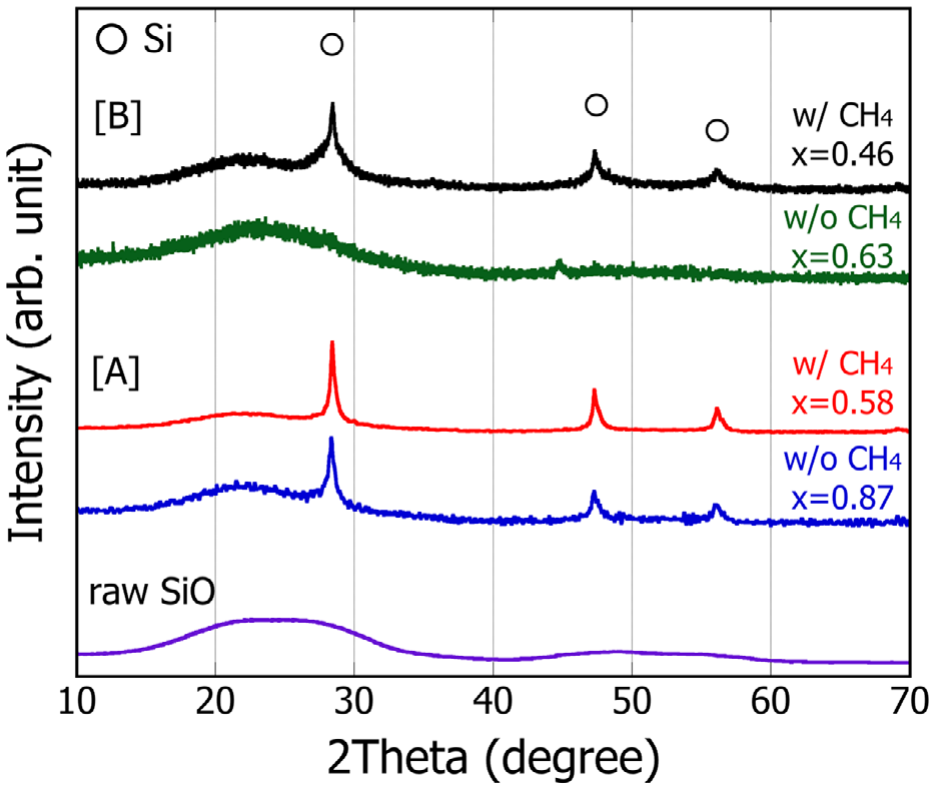
Comparison of XRD patterns of the SiO nanoparticles produced at different PS-PVD conditions. The estimated oxygen content of the SiO nanoparticles is also shown as *x* in the overall SiO_*x*_ representation.

Figure 1 shows the XRD patterns of the SiO nanoparticles processed at different PS-PVD conditions. The raw SiO powder shows only a broad pattern, indicating the presence of only the non-crystalline structure. Once this powder is processed under the condition [A], sharp peaks corresponding to the crystalline Si appear along with a broad pattern with slightly reduced intensity. With an addition of CH_4_, the diffraction patterns become sharper while the broad peak intensity decreases. For the case [B], in contrast, no diffraction patterns associated with the crystalline Si is observed and only the broad pattern is present for [B] without CH_4_. When CH_4_ is introduced during PS-PVD, crystalline Si phase is confirmed although the peak becomes rather broad, compared to that of the case [A], suggesting that Si particles become finer or partly amorphous.

**Figure 2.**
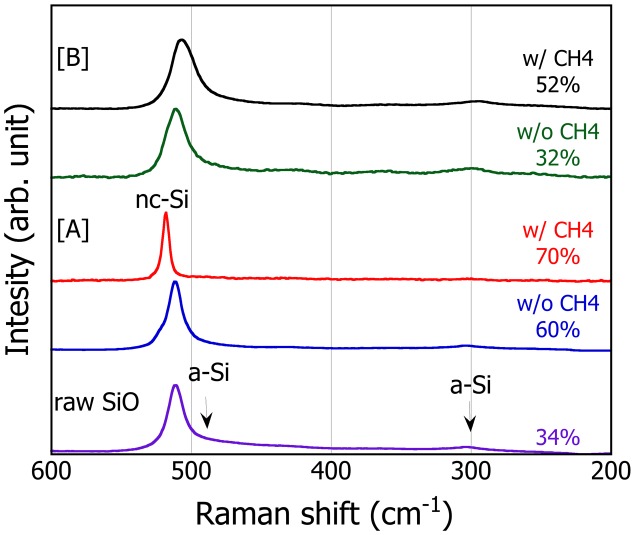
Raman scattering spectra for the SiO nanoparticles produced at different PS-PVD conditions. The estimated crystallinity of the nanoparticles is also shown for comparison.

Raman scattering spectra of these particles are shown in Figure 2. The peak associated with crystalline Si (c-Si) appears around 520 cm^−1^ for condition [A] while the corresponding peak for condition [B] seems to shift toward the lower wave number and become wider especially to the low wave number direction. Defining the crystallinity as the area fraction of a peak centered at 520 cm^−1^ (I_c_) with respect to the sum of I_c_ and the peak at 480 cm^−1^ (I_a_) associated with amorphous, quantitative analysis after peak separation reveals that the crystallinity for particles [A] is 60% (without CH_4_) and increases to 70% by addition of CH_4_, whereas that of the particle [B] is as little as 32% (without CH_4_) and reaches only 52% with CH_4_. These suggest that the condition [B] tends fundamentally to suppress crystallization and CH_4_ addition promotes crystallization for both conditions.

**Figure 3.**
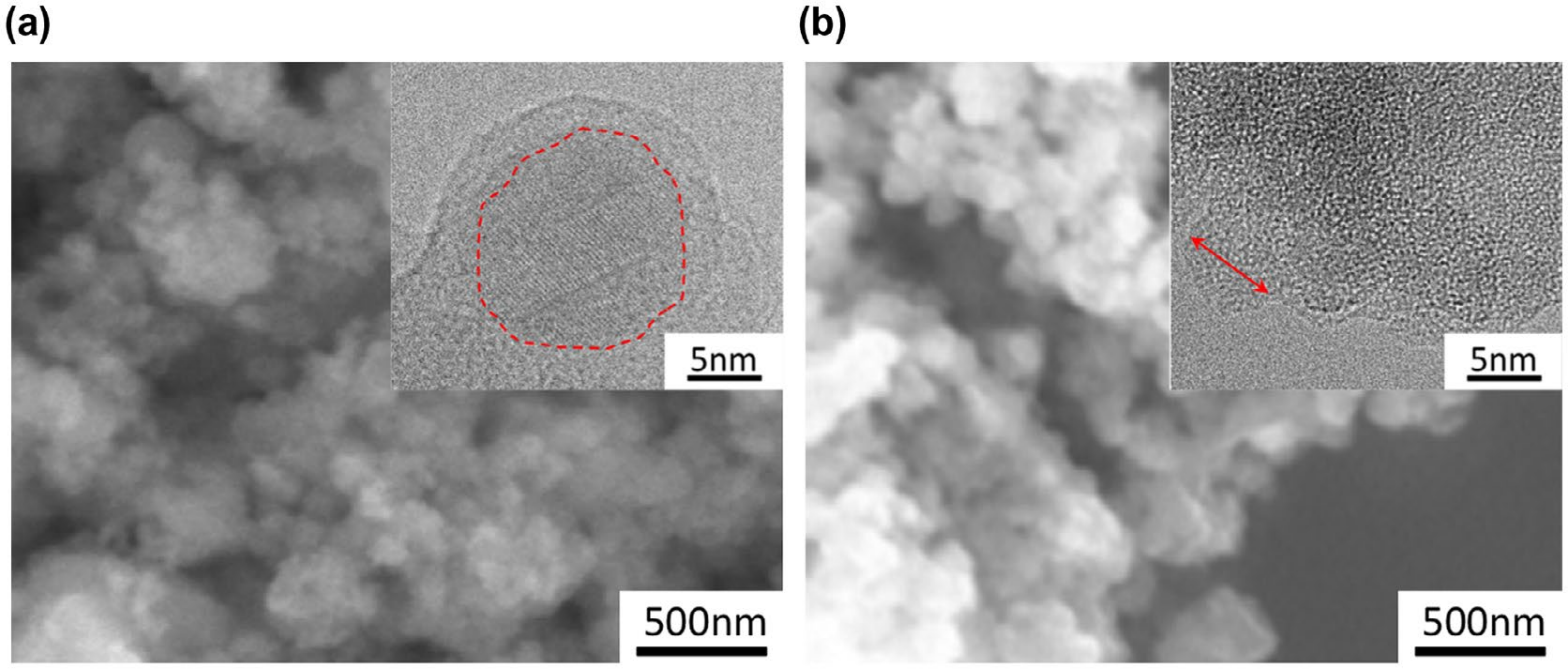
SEM images of the SiO particles processed by PS-PVD without CH_4_ addition under (a) [A] and (b) [B] condition. Inset is the STEM image of the primary particles. Circle and arrow are guide-to-eye for crystalline part and the primary particle, respectively.

The structure of these particles processed without CH_4_ is shown in Figure 3. From the SEM image, the particles at both [A] and [B] conditions are seen to form agglomerates of several 100 nm that are composed of smaller primary particles. In fact, TEM images (insets of Figure 3) show that the size of the primary particles is approximately 20 nm for case [A], whereas that for [B] looks slightly smaller, i.e. 10 nm on average. A major difference between these conditions is that a crystalline structure is surrounded by the amorphous shell for [A] while no lattice image is observed for [B]. This observation reasonably agrees with the presence/absence of the peaks in the XRD pattern shown in Figure 1.

The core-shell structure of [A] is suggestive of the occurrence of disproportionation reaction in a nanoparticle SiO.[27] Therefore, given that the XRD sharp and broad peaks are attributed to Si and SiO_2_, respectively, formed after disproportionation reaction of SiO, the degree of reduction of SiO can be quantified as the relative amount of oxygen, *x*, in the overall SiO_*x*_ representation by the phase content evaluation with the Rietveld analysis. Note that the value *x* so estimated is the maximum and may become even smaller if the shell is also partially reduced. As a result, *x* for the particles [A] are estimated to be 0.87 (with CH_4_) and 0.58 (without CH_4_), suggesting that the reduction of SiO powders is enhanced by an addition of CH_4_ during PS-PVD. In contrast, *x* of the particles [B] with CH_4_ is further reduced to be 0.46 compared to that of [A]. For the particles [B] without CH_4_, in which no appreciable Si peaks are observed in the XRD, *x* is estimated to be *x* = 0.63 by thermogravimetry analysis. This can be reasonably explained by the fact that the plasma gas temperature at condition [B] is expected to be higher than that at [A] and also that the CO(g) formation becomes more preferential to SiO at elevated temperatures.[26]. Therefore, the condition [B] works to promote reduction of SiO appreciably without CH_4_ as well as to make c-Si finer or to suppress crystalline phase formation. Unfortunately, it was hard to identify clearly the state of Si from electron energy loss spectra (EELS) and distinguish a-Si from a-SiO in this particle. Even so, as will be discussed in the next section, considering that the disproportionation reaction is promoted in a nanoparticle, it is plausible that the a-Si forms at the core at least partly as a result of disproportionation reaction at low temperature.[22]

**Figure 4.**
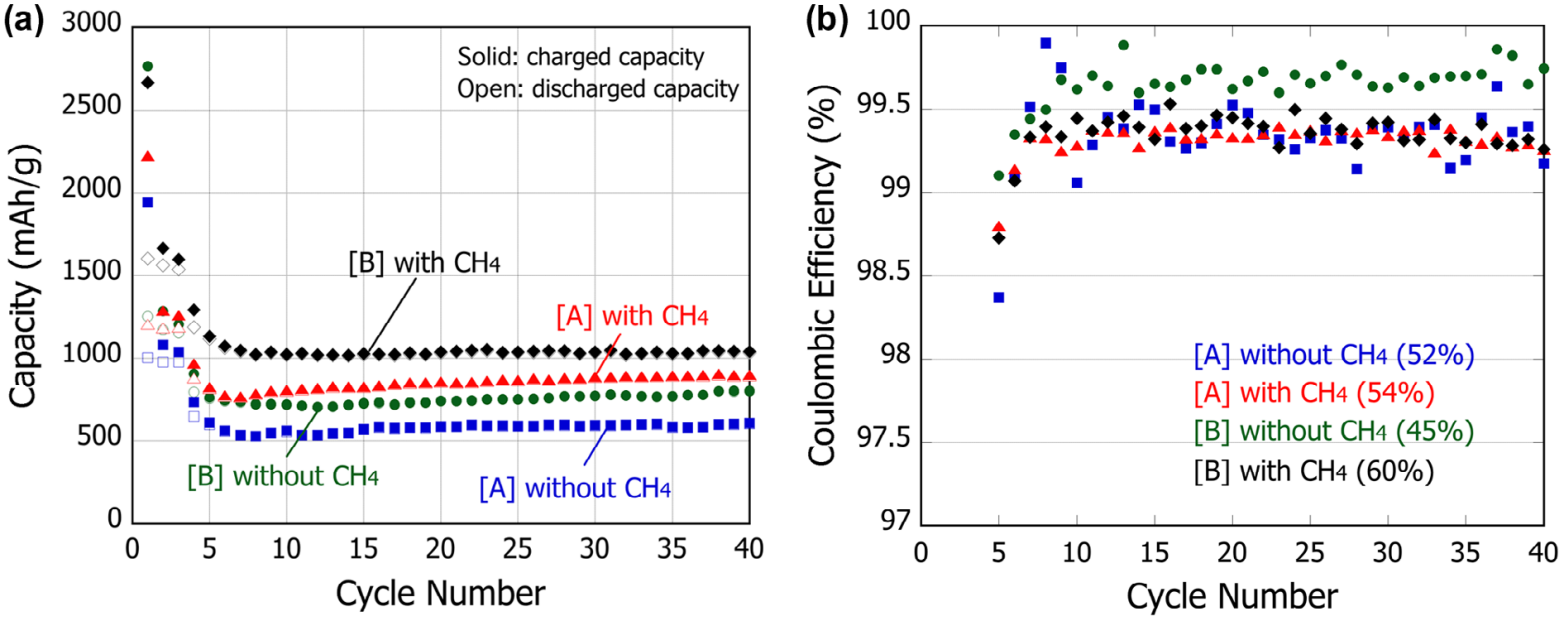
Comparison of the cycle capacity (a) and coulombic efficiency (b) of the PS-PVD SiO_x_ particles. Parenthesis in (b) shows the initial efficiency.

The half coin cells are assembled using these PS-PVD particles as anode and the cycle capacities and coulombic efficiencies are compared in Figure 4. As a general tendency, all the cells show a large capacity drop at the initial stage of the cycles but the capacities are stabilized after five cycles. For both cells with [A] and [B] particles, addition of CH_4_ is seen to increase the average capacity, and the initial capacity increases from 51.6% (without CH_4_) to 54.0% (with CH_4_) for [A] and 45.3% (without CH_4_) to 60.1% (with CH_4_) for [B]. In addition, irrespective of CH_4_ addition, the cells with the particles [B] show higher average capacity than that with [A] particles. These are reasonably explained by the increased degree of reduction, i.e. the increased Si relative volume. Moreover, considering also the fact that the fraction of c-Si in the [A] particles is relatively higher than that in [B], the reduction in the oxygen content has greater contributions to the increase in the capacity compared to the difference in the Si structure. Even so, it is interesting to note that the cell involving potentially a-Si ([B] without CH_4_) attains higher coulombic efficiency compared to other cells, and the a-Si structure at the pristine particle is therefore considered to be less vulnerable to the structural change in the repeated (de)lithiation. Nevertheless, the reduction during PS-PVD works effectively to compensate or even increase the inevitable low initial efficiency for nanosized particles.

### 3.2. Formation path of nanocomposite particles

#### 3.2.1. SiO nanoparticle growth

The premise of nanoparticle formation in PS-PVD is evaporation of the injected raw powders and the successive rapid condensation of the vapors so produced. Considering the fact that several 10 nm particles have been produced from 165 μm SiO powders under the condition [A] and also that the thermal load of powders at condition [B] is 0.275 of that at [A], the SiO vapor is potentially attained in the plasma under both conditions. The calculated equilibrium plasma chemistries also predict that SiO(g) is the most stable phase containing a Si atom and the relative amount of SiO(g) is somewhat constant at temperatures ranging from 2200 to 3500 K. Si(g) also forms because of decomposition of SiO but its relative amount is approximately smaller by 1/17 times than that of SiO(g) when no CH_4_ is added in the system.[27] Therefore, SiO(g) is considered to present as a major gas species in the plasma jet.

Girshick et al. [32] have suggested that nucleation from alloy vapors could be treated similarly to gas with a single element as long as the relevant chemical reactions are completed before the nucleation event^[32]^. SiO(s) is considered to form from SiO(g) vapor with no additional reactions during condensation. Therefore, homogeneous nucleation temperature of SiO(s) is estimated by the Becker-Döring classical nucleation model, taking into account the Lothe-Pound conception accordingly.[33,34] In the estimation, condensation of SiO(g) with constant gas partial pressure at 2500 K is assumed. Saturation vapor pressure of SiO reported by Ferguson and Nuth [35] and the temperature dependent specific surface energy of SiO(s) derived based on the scaled nucleation theory by Hale et al. [36] are also used. For the critical nucleation frequency, 10^17^ m^−3^s^−1^ is employed as is introduced in estimation of the surface energy.[36] As a result, homogeneous nucleation temperature of SiO (*T*_N(SiO)_) under [A] and [B] conditions is calculated to be 1584 and 1494 K, respectively, both of which are approximately 0.7–0.8 of the sublimation temperature of SiO (2070 K).[37]

**Figure 5.**
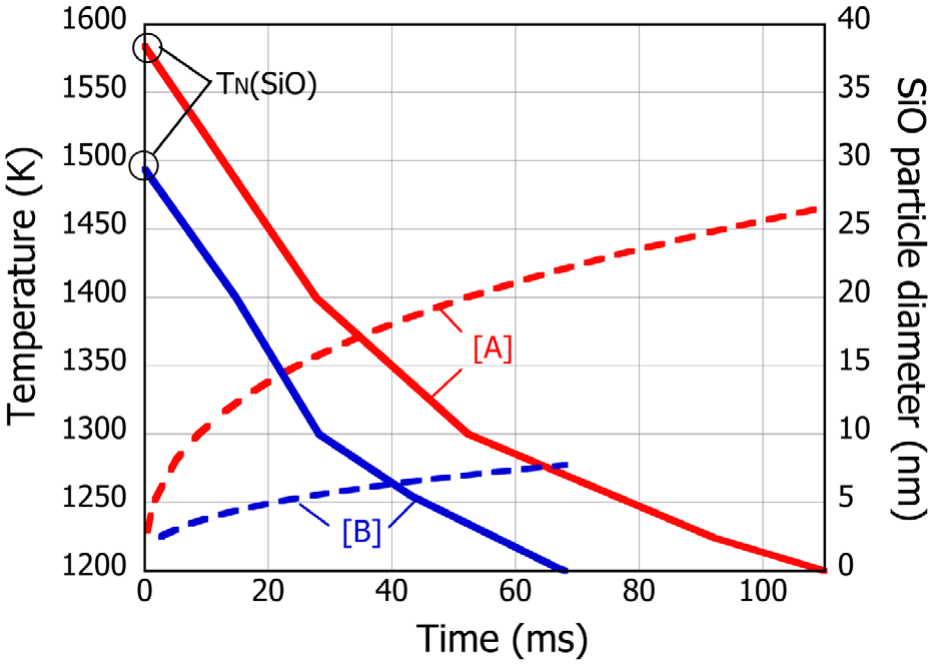
Typical cooling curves (solid) and the corresponding SiO particle size evolution (doted) for the PS-PVD condition [A] and [B].

Nanoparticle growth after homogeneous nucleation has been extensively discussed, and several models are widely known to predict quite nicely the experimental evidence.[32,38–40] In the present work, we employ rather simple model proposed by Ulrich,[40] which is confirmed to reproduce at least the growth of Si nanoparticles in PS-PVD.[30] Compared to the growth of Si, collision between SiO and H_2_ molecules in the plasma gas would become important as is found experimentally in the reduction of SiO in the Ar + H_2_ plasma. Therefore, the growth inhibition factor, which is the ratio of the collision frequency of SiO particles with respect to that between SiO and H_2_, is introduced in the SiO particle number density evolution, assuming that the SiO molecule that collides with H_2_ does not participate in the SiO growth. Moreover, the SiO particle would nucleate and grow along the gas stream in the vessel. Therefore, a typical gas streamline is selected from the simulated temperature and gas velocity distributions within the gas quenching vessel reported elsewhere,[31] and the SiO particle growth is calculated accordingly, as shown in Figure 5. The cooling curves below the estimated SiO homogeneous nucleation point are only shown in the figure. Not only the nucleation temperature but also the temperature of the gas stream within the vessel is lower for case [B]. Averaged cooling speed after nucleation for [B] is 4.5 × 10^3^ K s^–1^, which is slightly greater than 3.4 × 10^3^ K s^–1^ for [A]. With these ‘increased degrees of non-equilibrium effect’, the growth of SiO particle at condition [B] is suppressed although the particle is seen to grow monotonically for both conditions.

Meanwhile, fluid flow simulation has predicted that the surface of the vessel wall is at 1100 K for [A] and 1000 K for [B] on average, thus the nanoparticles attached on the wall would be exposed to the high temperature gases at this temperature for the processing time of 10 min at the longest. However, if one compares the calculated SiO particle size with the experimentally observed average particle size shown in Figure 3, i.e. 20 nm for [A] and 8 nm for [B], the SiO growth is expected to terminate at temperature around 1200–1300 K irrespective of the plasma conditions. That is, the SiO growth finishes during flight and the nanoparticles attached on the wall are readily subject to the disproportionation reaction at the wall temperature for the remaining PS-PVD time.

#### 3.2.2. Disproportionation reaction in a nanoparticle

It is known that SiO undergoes disproportionation reaction to form Si precipitates in SiO_2_ matrix when exposed to high temperature, and the size of precipitates varies significantly with annealing temperature and duration.[14,22,41,42] Generally, high temperature and long annealing time is required to form large Si precipitates: For instance, to attain crystalline Si precipitates as large as 10 nm, 4 h at 1423 K [41] or 24 min at 1573 K **[** 14] is necessary. When the annealing temperature is lowered below 1073 K, structure of the precipitates is primarily amorphous.[22,42] In this case, also, an annealing time as long as 18 h is required to produce 4.5 nm amorphous Si at 1073 K.

Formation of crystalline clusters through disproportionation reaction is considered to proceed by Si diffusion-controlled growth.[42] Although Nesbit [42] assumes that the diffusion coefficient is independent of the O/Si ratio, in the present work, adopting the diffusion-controlled disproportionation reaction model, oxygen content dependent diffusion coefficient, *D*(*x*), is introduced and estimated for bulk SiO using the data reported in the reference^[42]^. In this approach, the size of the precipitate *d*(*x*) is expressed as a function of annealing time *t* and temperature *T*:[41,43]

(1) $$d{\left(x\right)}^{2}-d_{0}^{2}=tD_{0}\left(x\right)\exp\left(-\frac{E_{a}}{kT}\right)\;$$

where *d*_0_ is the initial precipitate size, *E*_a_ the activation energy for diffusion, and *k* the Boltzmann constant. *D*_0_(*x*) is the *x* dependent pre-factor of diffusivity, i.e. *D*(*x*)=*D*_0_(*x*)exp(-*E*_a_/k*T*). Using the values *d*_0_ = 0.5 nm, *E*_a_ = 1.9 eV/atom and the Si precipitate size at a certain *x* and *T*, which are all reported by Nesbit, *D*_0_(*x*) is obtained as $D_{0}\left(x\right)=\left(4.35-2.36x\right){\times 10}^{-9}\left[{\text{cm}}^{2}/s\right]$

Meanwhile, with Equation (1), one can calculate the precipitate growth size ∆*d* during an infinitesimal time ∆*t* at a certain temperature *T*: the final Si precipitate size as a result of an annealing is thus given by summing ∆*d* for the entire thermal history of PS-PVD. Since the crystalline Si is potential to precipitate and grow at temperature higher than 1073 K below which amorphous Si is to form,[22] the disproportionation reaction in PS-PVD is assumed to start immediately after SiO nucleation and continue until PS-PVD finishes in 10 min. It is, therefore, noted that the size of the Si precipitate so estimated corresponds to the maximum size. Also, from Figure 3, the crystalline Si precipitate size *d* of the particles processed by PS-PVD is 20 nm on average and is much larger than *d*_0_, yielding $\left(d_{0}^{2}/d^{2}\right)<10^{-3}$. This reasonably allows us to ignore the term *d*_0_ in Equation (1). With these assumptions and the reported *E*_a_ of 1.9 eV/atom, Si precipitate size along the cooling history shown in Figure 5 is calculated to be approximately 0.5 nm, which is much smaller than the size observed in TEM shown in Figure 3. This large discrepancy is presumably due to the value *E*_a_ used which is derived for the bulk SiO not for nanoparticle.

To elucidate the characteristics of the disproportionation reaction in a nanosized SiO, the continuous-cooling-transformation (CCT) diagram is considered for formation of crystalline precipitates during cooling in PS-PVD. The nose of Si precipitates can be expressed by rearranging the equation (1):

(2) $$T=\frac{E_{a}}{k}{\left[\text{ln}\left(\frac{tD_{0}\left(x\right)}{d_{cr}{\left(x\right)}^{2}}\right)\right]}^{-1}$$

The critical precipitate size for crystallization *d*_cr_(*x*) and *E*_a_ can be uniquely determined by satisfying the experimental evidence that crystalline Si precipitate forms under condition [A] without CH_4_. That is, the cooling curve of [A] should intersect with the CCT nose of *x* = 0.87 and the c-Si core size *d* after cooling reaches as large as 15 nm, as is observed in Figure 3(a). To meet these conditions, *d*_cr_(*x*) is estimated to be 0.4 nm, which would be reasonable as the nucleation threshold for nanometer sized particle. Also, *E*_a_ is calculated to be 1.43 eV/atom, indicating that the less activation energy is required for diffusion in nanoparticle than bulk SiO.

**Figure 6.**
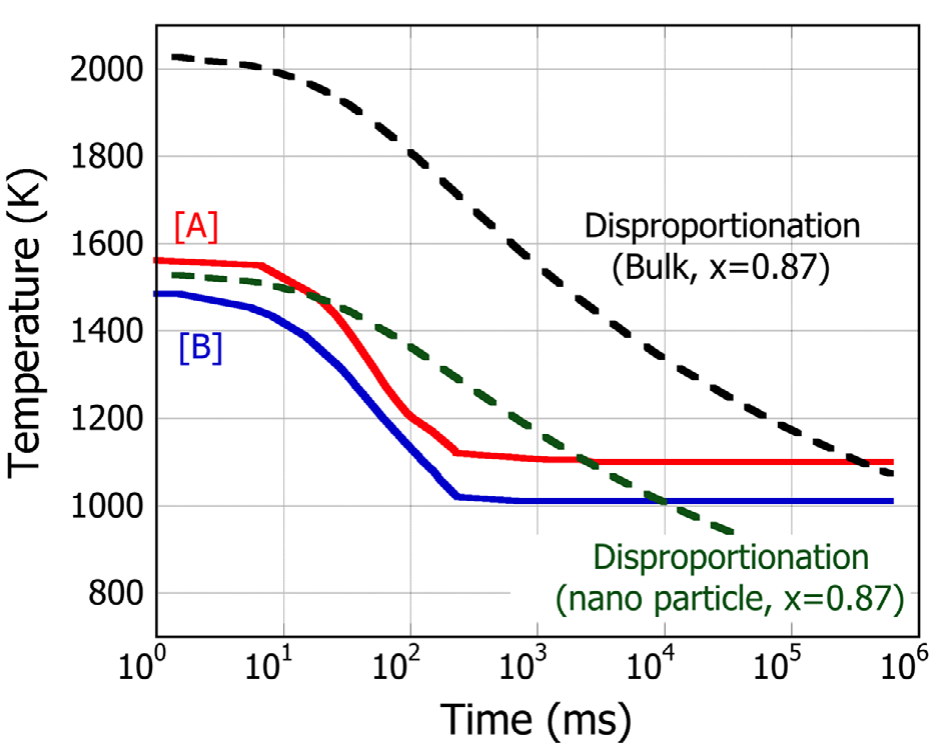
CCT diagram describing the nose of the disproportionation reaction of formation of crystalline Si precipitates (dotted line) both in bulk (black) and nanosized SiO_x_ (green) with *x* = 0.87. Cooling curves of case [A] and [B] are also shown for comparison.

The CCT nose estimated for nanoparticle SiO_x_ with *x* = 0.87 is shown in Figure 6 and compared with that of bulk, along with the cooling curves after the nucleation point of SiO for the cases [A] and [B]. It is seen that the CCT nose for bulk SiO intersects with the cooling curve of [A] only very close to the end of the processing (5 × 10^5^ ms). This implies that the c-Si hardly grows in a limited period during the processing and does not explain the c-Si formation in SiO nanoparticle observed in the experiment. In contrast, the CCT nose for SiO nanoparticle is estimated to shift towards lower temperatures. Although there are two intersects with the cooling of [A] at the elapsed time of 2 × 10^1^ and 2 × 10^3^ ms, the CCT nose at the SiO nucleation temperature (1584 K) is already below the cooling curve under condition [A], suggesting that disproportionation reaction occurs as soon as the SiO nanoparticle starts to grow. In the case of [B], on the other hand, despite that the CCT nose intersects with cooling curve at around 1 × 10^4^ ms, since the temperature drops immediately below 1073 K in 2 × 10^2^ ms, a-Si is expected to form before c-Si precipitates.

**Figure 7.**
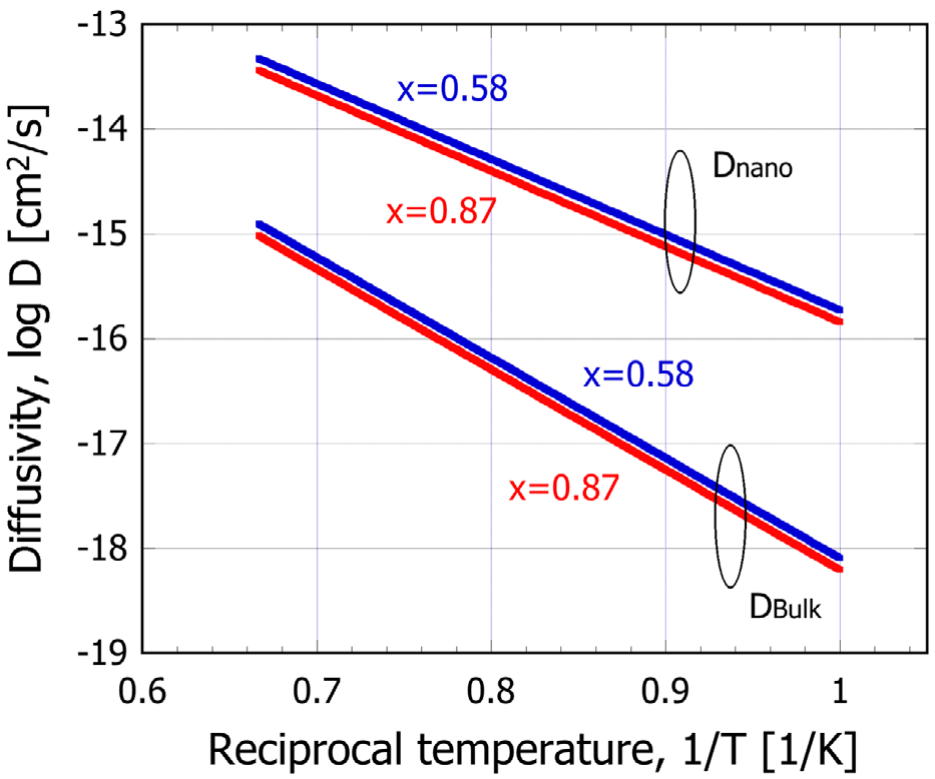
Variation of diffusion coefficient during disproportionation reaction of bulk and nanoparticle SiO_x = 0.58, 0.87_, as a function of the reciprocal temperature.

The estimated diffusivity *D*(*x*) is also plotted in Figure 7 as a function of temperature. It is clearly seen that diffusivity in nanoparticles increases by approximately two orders of magnitude than that for the bulk SiO within the temperature range considered in the present work. Also, diffusivity increases with decreasing the oxygen content x, although its effect is rather small compared to the particle size.

**Figure 8.**
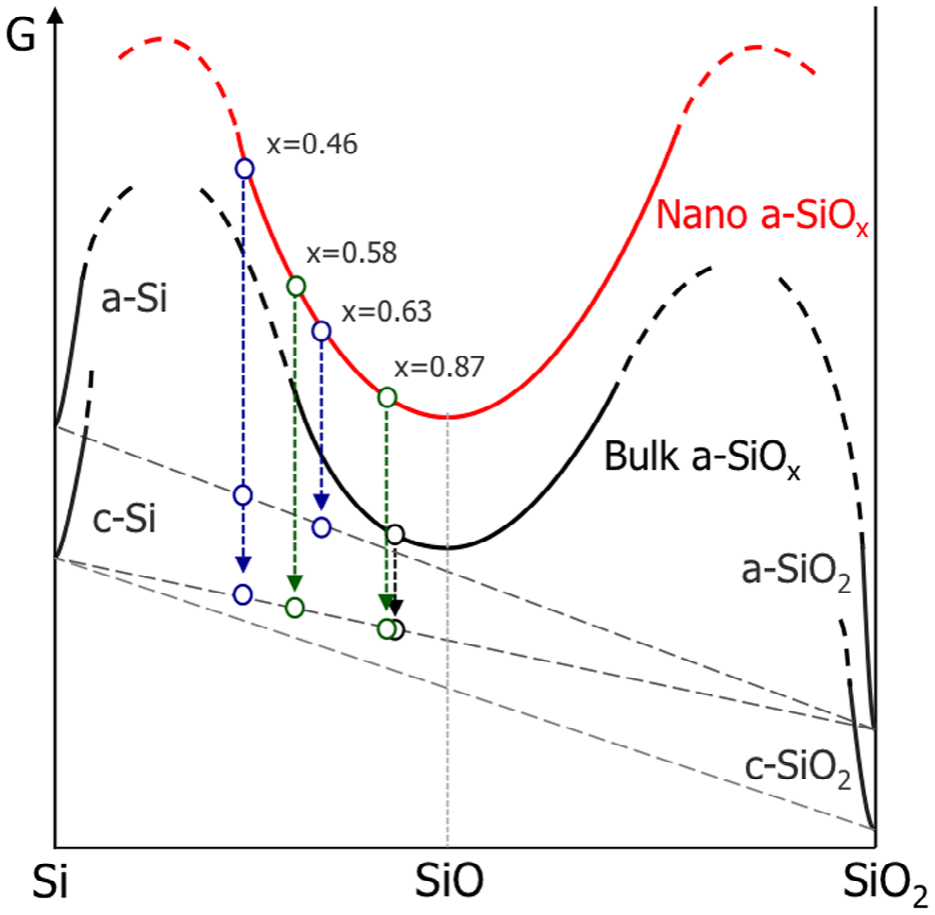
The Gibbs energy–composition diagram for the Si-O system.

Considering the phase and relative amount after disproportionation reaction of SiO_*x*_ with different *x*, the Gibbs energy–composition diagram for the Si-O system is drawn schematically in Figure 8. The Gibbs energy of amorphous SiO_*x*_ phase exists supposedly over a wide area of *x* having the local minimum at *x* = 1 as no other stable phase is reported in this system. Also, the Gibbs energy of SiO_*x*_ nanoparticle phase should be larger than that of bulk SiO_*x*_ phase as a result of the Gibbs–Thomson effect.

Under this relationship, when a-SiO_*x*_ is annealed at a certain temperature and disproportionation reaction takes place, the Gibbs energy of SiO_*x*_ phase with any *x* is dropped to the balanced energy of a mixture of a-SiO_2_ and either c-Si or a-Si, having the energy difference between the initial SiO_*x*_ and the a-SiO_2_ and a-Si/c-Si mixture as a driving force. It is therefore expected that greater driving force is attained for the nanoparticle and tends to promote the reaction. Also, this tendency should be further pronounced for SiO_x_ with smaller *x* due to the increased energy at *x* < 1. However, as seen in Figure 1, the nanoparticles processed under condition [B] (blue circle) include a small amount of c-Si (with CH_4_) or no c-Si (without CH_4_), suggesting that the corresponding energies of these nanoparticles are not necessarily on the same balanced energy line of the particles [A] (green circle). This readily supports that the disproportionation reaction is kinetically controlled, and the structure of the nanoparticle can be designed by the processing accordingly.

By adopting the same approach to the a-Si formation in bulk SiO reported in [22], the activation energy for amorphous phase formation *E*_a_^amo^ is estimated to be 0.14 eV/atom for *x* = 1, which is much smaller than 1.9 eV/atom for the c-Si in a bulk SiO. It is understood that a-Si formation proceeds with less energy because of lack of necessity of atomic alignment. In the case of nanoparticles, however, it was not possible to quantify the activation energy for a-Si formation in the nanoparticle [B] without CH_4_ because a-Si core was not distinguished from amorphous SiO shell clearly. Even so, considering that amorphous particle is produced in a short PS-PVD processing time, it is quite likely that the disproportionation reaction for a-Si formation is also promoted in a nanoparticle SiO.

## 4. Conclusions

Nanocomposite SiO_x_ particles 10–20 nm in diameter have been produced by PS-PVD. Addition of CH_4_ during PS-PVD is found to be effective in reducing SiO, which is further pronounced under the condition of less powder-feeding rate with greater gas-quenching degrees during PS-PVD. Under such environments, SiO particles are cooled rapidly to attain smaller size and the subsequent disproportionation reaction is also influenced to alter the particle structure from crystalline to amorphous. Batteries using these nanocomposites produced at the rapid-cooling condition with CH_4_ have exhibited increased cycle capacity and initial efficiency while maintaining stable cyclability. In addition, it is identified that Si diffusion during the disproportionation reaction is significantly enhanced in the nanosized particle, attaining greater diffusivity by two orders of magnitude than that in a bulk SiO, which facilitates instantaneous nanoparticle and composite structure formation in PS-PVD.
